# Sending repeat cultures: is there a role in the management of bacteremic episodes? (SCRIBE study)

**DOI:** 10.1186/s12879-016-1622-z

**Published:** 2016-06-13

**Authors:** J. Brad Wiggers, Wei Xiong, Nick Daneman

**Affiliations:** Department of Medicine, University of Toronto, Toronto, Canada; Sunnybrook Research Institute, Sunnybrook Health Sciences Centre, Toronto, Canada; Division of Infectious Diseases, Department of Medicine, Sunnybrook Health Sciences Centre, Toronto, Canada; Institute for Clinical Evaluative Sciences, Toronto, Canada; Division of Infectious Diseases & Clinical Epidemiology, Sunnybrook Health Sciences Centre, University of Toronto, Institute for Clinical Evaluative Sciences, 2075 Bayview Ave, G-wing Room 106, Toronto, M4N 3 M5 Canada

**Keywords:** Bacteremia, Bloodstream infection, Epidemiology, Blood cultures, Gram-positive bacteria, Gram-negative bacteria

## Abstract

**Background:**

In the management of bacteremia, positive repeat blood cultures (persistent bacteremia) are associated with increased mortality. However, blood cultures are costly and it is likely unnecessary to repeat them for many patients. We assessed predictors of persistent bacteremia that should prompt repeat blood cultures.

**Methods:**

We conducted a retrospective cohort study of bacteremias at an academic hospital from April 2010 to June 2014. We examined variables associated with patients undergoing repeat blood cultures, and with repeat cultures being positive. A nested case control analysis was performed on a subset of patients with repeat cultures.

**Results:**

Among 1801 index bacteremias, repeat cultures were drawn for 701 patients (38.9 %), and 118 persistent bacteremias (6.6 %) were detected. Endovascular source (adjusted odds ratio [aOR], 7.66; 95 % confidence interval [CI], 2.30-25.48), epidural source (aOR, 26.99; 95 % CI, 1.91-391.08), and *Staphylococcus aureus* bacteremia (aOR, 4.49; 95 % CI, 1.88-10.73) were independently associated with persistent bacteremia. *Escherichia coli* (5.1 %, *P* = 0.006), viridans group (1.7 %, *P* = 0.035) and β-hemolytic streptococci (0 %, *P* = 0.028) were associated with a lower likelihood of persistent bacteremia. Patients with persistent bacteremia were less likely to have achieved source control within 48 h of the index event (29.7 % vs 52.5 %, *P* < .001), but after variable reduction, source control was not retained in the final multivariable model.

**Conclusions:**

Patients with *S. aureus* bacteremia or endovascular infection are at risk of persistent bacteremia. Achieving source control within 48 h of the index bacteremia may help clear the infection. Repeat cultures after 48 h are low yield for most Gram-negative and streptococcal bacteremias.

## Background

Blood cultures are common investigations in the assessment of a broad range of infectious syndromes, detecting approximately 200,000 cases of bacteremia annually in the United States [1]. Despite such high incidence, studies have highlighted the low yield of blood cultures in a number of clinical settings, including cellulitis [2], community-acquired pneumonia [3, 4], pyelonephritis [5, 6], and isolated fever or leukocytosis [1]. Although the utility in these settings is debatable, there is evidence of ongoing unrestrained blood culture use [7].

For confirmed cases of bacteremia, repeat cultures are recommended for *Staphylococcus aureus* bacteremia and infective endocarditis [8, 9]. Breakthrough bacteremia occurs in approximately 6 % of bacteremic episodes and is an independent predictor of death [10]. A study of *Klebsiella pneumoniae* bacteremia showed that repeat cultures were drawn in 81 % of cases despite only a 7 % incidence of persistent bacteremia, and suggested that a clinical scoring system could be applied to decide which patients warranted repeat cultures [11]. Data to guide the use of repeat cultures for other organisms is lacking, despite a “conventional wisdom” that they should be avoided [8, 9].

At Sunnybrook Health Sciences Centre (SHSC), approximately 20,000 sets of blood cultures are processed annually. In the current climate of soaring health care costs, movements such as the *Choosing Wisely* campaign are encouraging physicians to rethink the use of common investigations with questionable value [12]. Given that the overall yield of blood cultures is approximately 4–7 % [1], the use of initial and repeat blood cultures should continue to be scrutinized.

We evaluated the current patterns of repeat blood culture use at our institution and the epidemiology of persistent bacteremia. Our goal was to examine clinical and microbiologic variables that were associated with persistent bacteremia, and thereby identify bacteremic episodes with high yield and low yield for repeat culture use.

## Methods

### Study design, setting and patients

We conducted a retrospective cohort study of adult patients admitted to SHSC between April 1 2010 and June 30 2014 with a first episode of bacteremia. Patients aged <17 years were excluded. Subsequent bacteremic episodes for the same patient were also excluded. A nested case control study was also conducted, comparing individuals with persistent bacteremia to a randomly sampled subset of patients with cleared bacteremia.

SHSC is an academic hospital in Toronto, Canada that services approximately 31,000 patients annually, with 824 acute care beds including 82 intensive care beds. The study was approved by the SHSC ethics review board.

### Data sources

Patients with episodes of bacteremia were identified using the Stewardship Program Integrated Resource Information Technology (SPIRIT) database at SHSC. The database, as previously described [13, 14], is automatically populated by health level 7 (HL7) messages from pharmacy, microbiology and electronic patient care databases for all admitted and previously admitted patients. For the patients included in the nested case control analysis, a focused review of the patients’ charts was conducted to extract more detailed clinical data regarding the source of infection and the patients’ clinical status at the time of repeat blood culture collection.

### Blood culture collection, processing and reporting

Blood cultures at SHSC are performed using BACTEC 9240 blood culture system (Becton Dickinson Diagnostic Instruments Systems, USA) in accordance with Clinical Laboratory Standards Institute guidelines [9]. A blood culture set consists of blood collected into one aerobic and one anaerobic culture bottle, each receiving 10 mL of blood. The number of sets collected was determined by the ordering physician as part of usual clinical care. Positive cultures are Gram stained and then plated on blood agar, chocolate agar, colistin-nalidixic acid agar, MacConkey agar and brucella agar plates. Gram-negative colonies are identified by VITEK® 2 (model 510731-9EN1, bioMérieux, Inc., USA), while Gram-positive colonies are identified by various spot techniques depending on their morphology and configuration (latex agglutination, tube coagulase, StaphSR, etc.).

### Definitions

A clinically significant bacteremia was defined as at least one positive blood culture set with a bacterium that is not part of the commensal skin flora. Cultures with coagulase-negative staphylococci, Diphtheroids, or *Bacillus* spp. were individually assessed to determine whether they were clinically significant or contaminants. Criteria included clinical evidence of sepsis, multiple positive blood culture sets, or evidence of primary site of infection with the same organism [8]. *Polymicrobial bacteremia* was defined as 2 or more clinically significant bacterial species isolated from the same blood culture set. The bacteremia was considered *hospital-acquired* if the blood culture was drawn more than 48 h after the admission time.

The *index bacteremia* was defined as the first clinically significant bacteremia that occurred for a patient during our study window. We first determined whether or not any repeat blood cultures were sent in the interval between 2–7 days after the index bacteremia. If at least one repeat blood culture set drawn 2–7 day after the index bacteremia grew the same organism, the event was defined as a *persistent bacteremia*. When all repeat cultures in this window were negative, this was defined as a *cleared bacteremia*. For polymicrobial index bacteremias, persistent bacteremia was defined as detection of any one or more of the index pathogens from a repeat culture during the 2–7 day window. Repeat cultures drawn in the first 48 h were excluded from these analyses, because (1) multiple blood culture sets are often sent as part of an initial diagnostic work-up, (2) within 48 h the clinical team would often not yet be aware of the positivity of the original culture, and (3) this narrow time interval would be too brief to qualify as persistent bacteremia.

Infections were defined broadly as having an endovascular, extravascular or unknown source. *Endovascular infections* in this study included infective endocarditis, infected central venous catheter, pacemaker infection and vascular graft infection. *Extravascular infections* were those with a focus identified in tissue, organ or body cavity based on the clinical presentation and the results of imaging, culture or biopsy investigations. *Unknown source* was the designation for those bacteremias for which no source could be identified based on clinical presentation and standard investigations.

*Surveillance cultures* were defined as repeat cultures drawn to document blood sterility after the initiation of antibiotics, or those drawn in the absence of clinical variables of instability (systemic inflammatory response syndrome (SIRS) criteria, isolated fever (T > 38.0 °C), or leukocytosis (WBC > 12,000)).

### Covariates

Variables of interest collected in this study included: patient demographics (age, sex and comorbidities), hospital variables (admitting service), culture variables (date and time of blood culture collection, identities and susceptibilities of isolates, timing and results of subsequent cultures), infectious syndrome variables (site of primary infection), patient variables (vital signs, leukocyte count and physician impression at time of repeat culture as documented in progress notes), treatment variables (timing of adequate antibiotic therapy, need and timeliness of source control), and outcome variables (ICU admission within 72 h of index bacteremia, length of stay and mortality). For mortality, because inclusion into the cohort with repeated blood cultures necessitated being alive at least 48 h after the index bacteremia, 7-day and 30-day mortality actually represent mortality between 2–7 days and 2–30 days, respectively. For those without repeat blood cultures, 7-day mortality had the same definition, but 30-day mortality represented 0–30 day mortality.

### Statistical analysis

**Fig. 1:**
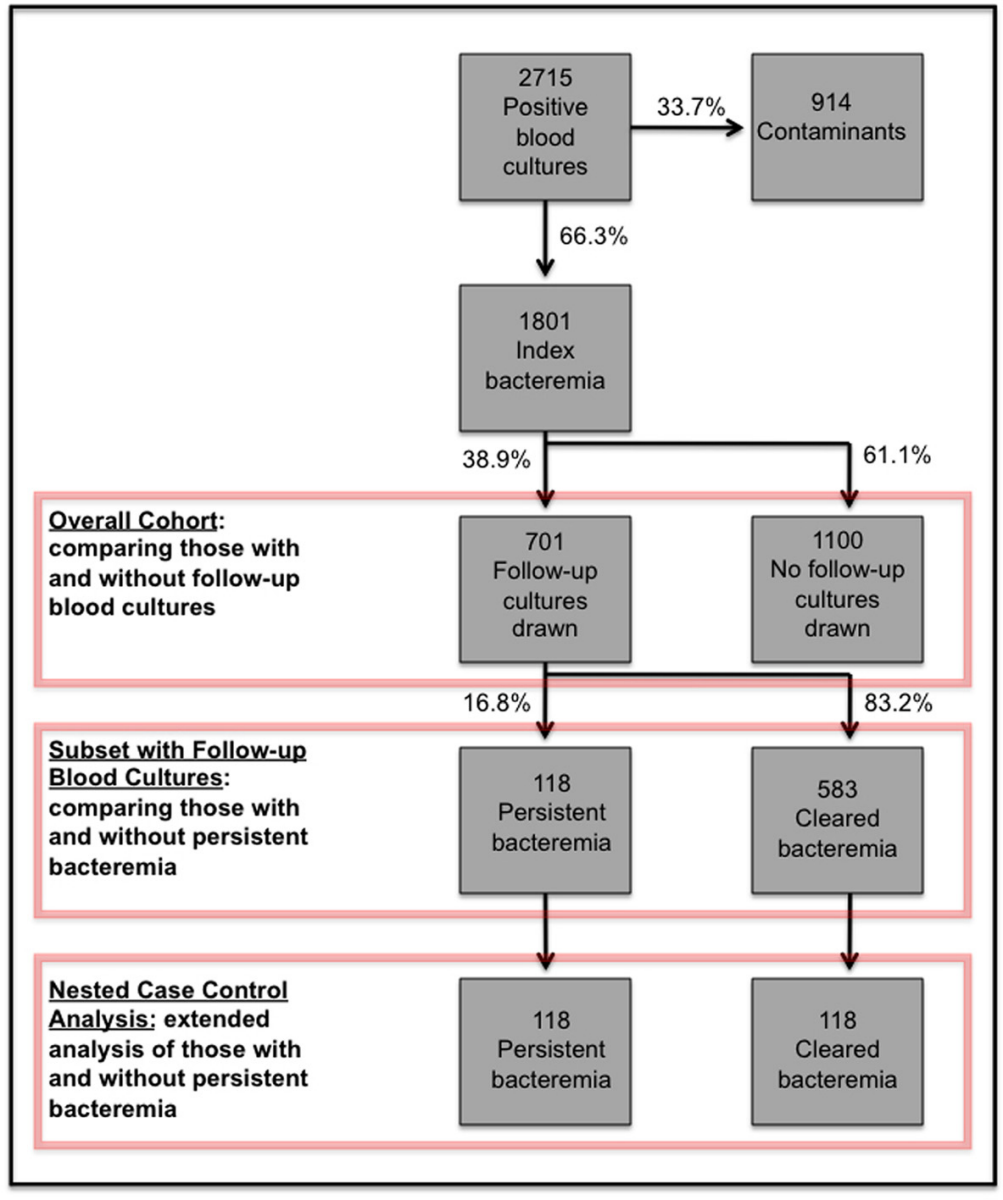
Distribution of patients in the cohort and nested case control analyses, according to receipt of repeat blood culture testing, and documentation of persistent versus cleared bacteremia. NOTE: The 118 cleared bacteremia included in the nested case control analysis were randomly sampled from the full cohort

First, we compared patient and pathogen characteristics among patients who did or did not undergo repeat blood culture testing. Next, we assessed only those patients who had repeat blood cultures drawn. We compared the probability that, for a given covariate (i.e. *E. coli* as the causative organism), a repeat culture drawn 2–7 days after the index bacteremia would be positive (persistent bacteremia) or negative (cleared bacteremia). Lastly, we performed a nested case control study, comparing patients with persistent bacteremia to an equally large subset of randomly sampled patients with cleared bacteremia, once again assessing the association between covariates and whether the repeat cultures were positive or negative. Please refer to the flow chart in Fig. 1 for an overview of the different comparator groups and their sizes. Categorical variables were compared using the Chi Square test or Fisher’s exact test when necessary. Continuous variables were compared using the Wilcoxon rank sum test. Multivariable logistic regression was performed including all variables associated with persistent bacteremia in bivariate analyses in the nested case control analysis, with stepwise backward selection for variable reduction until all retained variables were statistically significant at *p*-value threshold <0.05.

We calculated the number of avoidable repeated blood cultures obtained among those with no documentation of clinical instability or deterioration, and no high yield patient or pathogen characteristics for persistent bacteremia. We calculated a cost range using values reported in the literature that were felt to approximate our institution, with different cost for negative ($15–50) and positive ($67–100) cultures [15, 16]. All analyses were performed using Microsoft Excel or SAS software (version 9.3; SAS institute).

## Results

There were a total of 2715 patients with positive blood cultures (Fig. 1). Of these, 914 (33.7 %) were contaminants, leaving 1801 index bacteremias included in our cohort. Repeat cultures were drawn for 701 (38.9 %) patients between 2–7 days from the index event. The repeat culture demonstrated a persistent bacteremia in 118 patients (6.6 %). Of the 583 cleared bacteremias, 118 were randomly sampled for inclusion in the nested case control analysis (Fig. 1).

### Cohort study

#### Admission service

Although repeat cultures were more common among surgical in-patients (44.2 % vs 35.5 %, *p =* 0.003), these individuals were statistically more likely to have a cleared bacteremia (46.7 % vs 32.2 %, *p =* 0.004) (Table 1). In contrast, repeat cultures were less likely to be done on medical in-patients (45.0 % vs 37.9 %, *p* < .001), but persistent bacteremia was more common in this group (55.1 % vs 34.5 %, *p* < .001) (Table 1).

**Table 1:** Characteristics of patients with bacteremia according to whether repeat cultures were obtained, and whether repeat cultures were persistently positive

	Overall cohort (*n* = 1801)			Subset with repeat blood cultures (*n* = 701)		
	No repeat cultures (*n* = 1100)	Repeat cultures (*n* = 701)	*P*	Cleared bacteremia (*n* = 583)	Persistent bacteremia (*n* = 118)	*P*
Male n(%)	576 (52.4)	442 (63.1)	<**0.001**	361 (61.9)	80 (67.8)	0.23
Age(y) median (IQR)	71 (56.5–82)	66 (51–77)	<**0.001**	65 (51–77)	68 (52–80)	0.26
Length of stay(d) median (IQR)	12 (5–30)	26 (12–48)	<**0.001**	26 (12–50)	25 (12–41)	0.59
Hospital-acquired n(%)	514 (46.7)	389 (55.5)	<**0.001**	333 (57.1)	55 (46.6)	**0.036**
Admitting service n(%)						
Medical	495 (45.0)	266 (37.9)	**0.003**	201 (34.5)	65 (55.1)	<**0.001**
Surgical	390 (35.5)	310 (44.2)	<**0.001**	272 (46.7)	39 (33.1)	**0.007**
Heme-Onc	215 (19.5)	125 (17.8)	0.36	110 (18.9)	14 (11.9)	0.07
Organism n(%)						
Total Gram-positive bacteria	425 (38.6)	457 (65.2)	<**0.001**	367 (63.0)	90 (76.3)	**0.006**
*S. aureus*	97 (8.8)	162 (23.1)	<**0.001**	108 (18.5)	53 (44.9)	<**0.001**
Skin commensals	62 (5.6)	114 (16.3)	<**0.001**	98 (16.8)	16 (13.6)	0.38
*Enterococcus* spp.	110 (10.0)	102 (14.6)	**0.004**	82 (14.1)	20 (16.9)	0.42
*S. pneumoniae*	22 (2.0)	11 (1.6)	0.506	11 (1.9)	0	0.13
Viridans group streptococci	48 (4.4)	41 (5.9)	0.156	39 (6.7)	2 (1.7)	**0.031**
β-hemolytic streptococci	46 (4.2)	23 (3.3)	0.33	23 (4.0)	0	**0.021**
*S. anginosus* group	31 (2.8)	11 (1.6)	0.087	10 (1.7)	1 (0.9)	0.70
Other Gram-positive bacteria	35 (3.2)	30 (4.3)	0.22	29 (5.0)	1 (0.9)	**0.044**
Total Gram-negative bacteria	654 (59.4)	247 (35.2)	<**0.001**	220 (37.7)	27 (22.9)	**0.002**
*E. coli*	329 (29.9)	90 (12.8)	<**0.001**	84 (14.4)	6 (5.1)	**0.006**
*Klebsiella* spp.	143 (13.0)	43 (6.1)	<**0.001**	33 (5.7)	10 (8.5)	0.25
*Pseudomonas* spp.	45 (4.1)	32 (4.6)	0.63	27 (4.6)	5 (4.2)	0.85
AmpC-producers	102 (9.3)	59 (8.4)	0.54	53 (9.1)	6 (5.1)	0.15
Other Gram-negative bacteria	69 (6.3)	39 (5.6)	0.54	35 (6.0)	4 (3.4)	0.26
Anaerobes	86 (7.8)	34 (4.9)	**0.014**	32 (5.5)	2 (1.7)	0.1
Polymicrobial n(%)	107 (9.7)	75 (10.7)	0.51	66 (11.3)	9 (7.6)	0.24
Mortality n(%)						
2-day	97 (8.8)	-	-	-	-	-
7-day	44 (4.0)	42 (6.0)	0.053	29 (5.0)	13 (11.0)	**0.012**
30-day	123 (11.2)	189 (27.0)	<**0.001**	160 (27.4)	29 (24.6)	0.52

*NOTE: P* values were calculated using the Chi square or Fisher’s exact test for categorical variables and Wilcoxon rank sum test for continuous variables. *P* value threshold for significance was <0.05 (statistically significant values are bolded). *IQR* interquartile range, *Heme-Onc* hematology-oncology in-patient ward. Skin commensals include coagulase-negative staphylococci, *Bacillus* spp., Diphtheroids. AmpC-producers include *Serratia marcescens, Providencia stuartii, Proteus vulgaris, Citrobacter* spp., *Enterobacter* spp., *Morganella morganii*

#### Microbiology

**Fig. 2:**
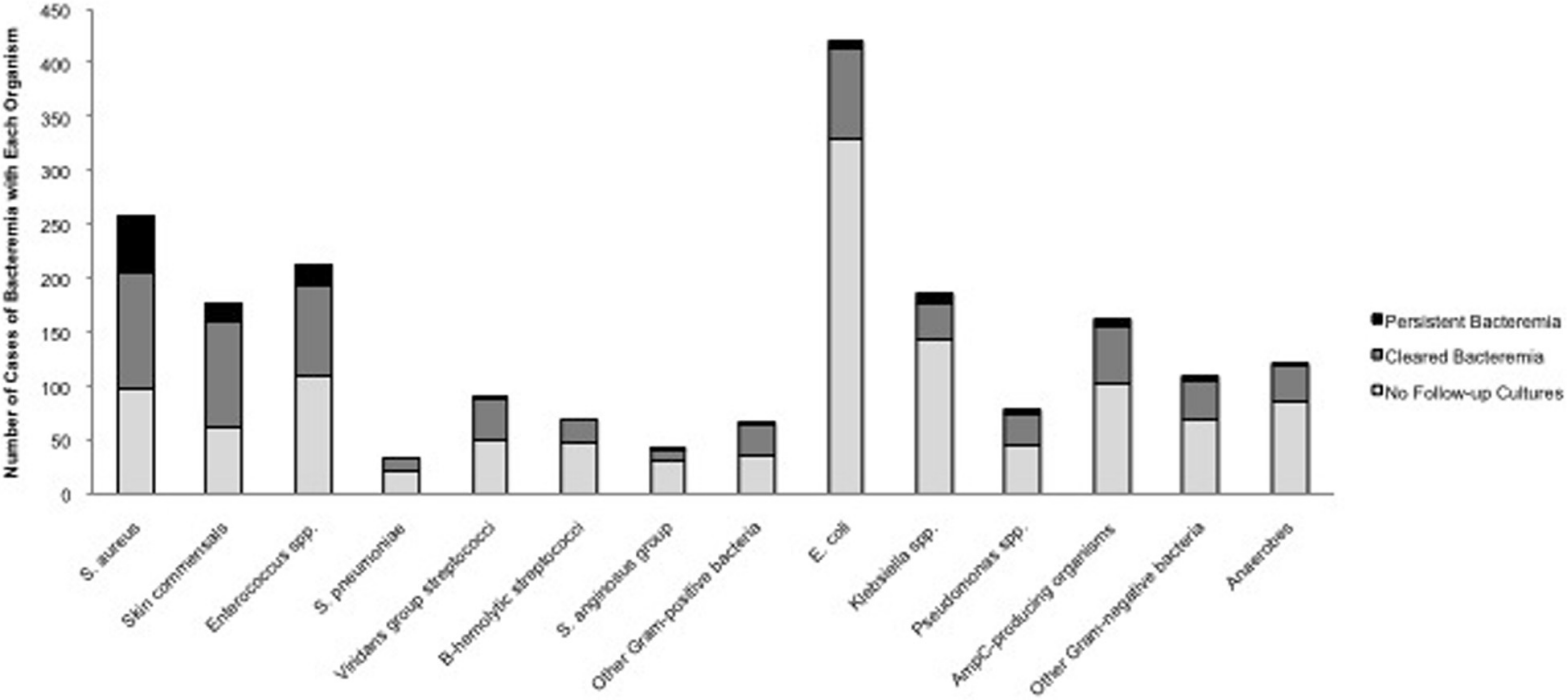
Distribution of organisms causing bacteremia and persistent bacteremia. NOTE: Skin commensals include coagulase-negative staphylococci, *Bacillus* spp., Diphtheroids. AmpC-producers include *Serratia marcescens, Providencia stuartii, Proteus vulgaris, Citrobacter* spp., *Enterobacter* spp., *Morganella morganii*

*Escherichia coli* was the most common cause of index bacteremia (23.3 %) (Fig. 2). The most common cause of persistent bacteremia was *S. aureus* (45.8 %), followed by *Enterococcus* spp. (16.9 %) (Table 1). *E. coli* was a rarer cause of persistent bacteremia (5.1 % of persistent bacteremias, and 1.4 % of cases of index bacteremias caused by this organism) (Fig. 2).

Repeat cultures were more commonly drawn for Gram-positive organisms, particularly *S. aureus* (23.1 % vs 8.8 %, *p* < .001) (Table 1). These organisms also had an increased likelihood of causing persistent bacteremia (45.8 % vs 18.5 %, *p* < .001) (Table 1). Among the various *Streptococcus* spp., there was no difference in whether or not repeat cultures were drawn, however β-hemolytic and viridans group streptococci were more likely to have cleared when repeat cultures were drawn (Table 1). Gram-negative organisms were less likely to have repeat cultures drawn (32.2 % vs 59.4 %, *p* < .001), and *E. coli* was significantly associated with a cleared bacteremia (14.4 % vs 5.1 %, *p =* .006) (Table 1).

#### Outcomes

The median length of stay was longer for those with repeat cultures compared to those without (26 vs 12 days, *p* < .001) (Table 1). Individuals who underwent repeat cultures had 27 % mortality at 30 days (Table 1). This was significantly higher than those with no repeat cultures (11.2 %, *p* < .001) (Table 1). There was no difference in 30-day mortality between patients with cleared and persistent bacteremia (Table 1).

### Nested case control analysis

#### Population characteristics

There was no significant difference in any comorbidities between individuals with cleared or persistent bacteremia (Table 2). With respect to admission service, the association between persistent bacteremia and admission to a medical in-patient service remained significant in the nested case control analysis (55.1 % vs 36.4 %, *p =* 0.004) (Table 2). Trends in microorganism distribution between cleared and persistent bacteremia were largely preserved. *S. aureus* was associated with persistent bacteremia, while cleared bacteremia was associated with *E. coli*, *Streptococcus pneumoniae*, β-hemolytic and viridans group streptococci (Table 2).

**Table 2:** Nested case control analysis: characteristics of patients with cleared versus persistent bacteremia

	Cleared bacteremia (*n* = 118)	Persistent bacteremia (*n* = 118)	*P*
Male n (%)	70 (59.8)	80 (67.8)	0.18
Age(y) median (IQR)	66.5 (54–77)	68 (52–80)	0.61
Comorbidities n (%)			
Cardiac	41 (34.7)	45 (38.1)	0.59
Respiratory	10 (8.5)	6 (5.1)	0.30
Liver	7 (5.9)	3 (2.5)	0.22
Diabetes	24 (20.3)	34 (28.8)	0.13
Dialysis	8 (6.8)	13 (11.0)	0.25
Solid tumor	33 (28.0)	24 (20.3)	0.17
Hematologic malignancy	14 (11.9)	7 (5.9)	0.11
Burn	6 (5.1)	6 (5.1)	1.0
Polytrauma	13 (11.0)	11 (9.3)	0.67
HIV	1 (0.9)	1 (0.9)	1.0
Neutropenia	15 (12.7)	8 (6.8)	0.12
Length of stay(d) median(IQR)	24 (13–52)	25 (12–41)	0.44
Admitting service n(%)			
Medical	43 (36.4)	65 (55.1)	**0.004**
Surgical	51 (43.2)	39 (33.1)	0.11
Heme-Onc	24 (20.3)	14 (11.9)	0.71
ICU within 72 h of bacteremia n(%)	44 (37.3)	39 (33.1)	0.54
Hospital-acquired n (%)	58 (49.2)	55 (46.6)	0.70
Polymicrobial n(%)	21 (17.8)	9 (7.6)	**0.019**
Organism n(%)			
Total Gram-positive bacteria	77 (65.3)	90 (76.3)	**0.044**
*S. aureus*	26 (22.0)	53 (44.9)	<**0.001**
Skin commensals	10 (8.5)	16 (13.6)	0.29
*Enterococcus* spp.	23 (19.5)	20 (16.9)	0.50
*S. pneumoniae*	5 (4.2)	0	**0.03**
Viridans group streptococci	11 (9.3)	2 (1.7)	**0.011**
β-hemolytic streptococci	5 (4.2)	0	**0.03**
*S. anginosus* group	3 (2.5)	1 (0.9)	0.31
Other Gram-positive bacteria	6 (5.1)	1 (0.9)	0.062
Total Gram-negative bacteria	44 (37.3)	27 (22.9)	**0.016**
*E. coli*	16 (13.6)	6 (5.1)	**0.048**
*Klebsiella* spp.	8 (6.8)	10 (8.5)	0.62
*Pseudomonas* spp.	6 (5.1)	5 (4.2)	0.76
AmpC-producing bacteria	12 (10.2)	6 (5.1)	0.14
Other Gram-negative bacteria	6 (5.1)	4 (3.4)	0.54
Anaerobic bacteria	5 (4.2)	2 (1.7)	0.28
7-day mortality n(%)	5 (4.2)	13 (11.0)	**0.05**
30-day mortality n(%)	13 (11.0)	29 (24.6)	**0.006**

*NOTE: P* values were calculated using the Chi square or Fisher’s exact test for categorical variables and Wilcoxon rank sum test for continuous variables. *P* value threshold for significance was <0.05 (statistically significant values are bolded). *IQR* interquartile range, *HIV* human immunodeficiency virus, *ICU* intensive care unit, *Heme-Onc* hematology-oncology in-patient ward. Skin commensals include coagulase-negative staphylococci, *Bacillus* spp., Diphtheroids. AmpC-producers include *Serratia marcescens, Providencia stuartii, Proteus vulgaris, Citrobacter* spp., *Enterobacter* spp., *Morganella morganii*

#### Clinical syndrome

Endovascular infections were statistically associated with persistent bacteremia (44.1 % vs 23.7 %, *p* < .001), largely driven by central catheter and intravascular device infections (Table 3). Among extravascular syndromes, epidural abscesses and discitis were associated with persistent bacteremia, while genito-urinary infections were more commonly associated with cleared bacteremia (Table 3).

**Table 3:** Nested case control analysis of persistent versus cleared bacteremia: clinical syndrome and clinical parameters at the time of repeat blood culture collection

	Cleared bacteremia (*n* = 118)	Persistent bacteremia (*n* = 118)	*P*
Clinical Syndrome n(%)			
Endovascular	28 (23.7)	52 (44.1)	<**0.001**
Endocarditis	9 (7.6)	18 (15.3)	0.066
Central line/device/graft	19 (16.1)	34 (28.8)	**0.019**
Extravascular	68 (57.6)	54 (45.8)	0.068
Abdominal	19 (16.1)	10 (8.5)	0.074
Cardio-respiratory	18 (15.3)	11 (9.3)	0.17
Genito-urinary	20 (16.9)	7 (5.9)	**0.008**
SSTI	9 (7.6)	15 (12.7)	0.20
Epidural abscess/discitis	1 (0.9)	7 (5.9)	**0.036**
Septic arthritis	1 (0.9)	5 (4.2)	0.121
Unknown	22 (18.6)	12 (10.2)	0.064
Clinical parameters at time of repeat culture [present/total (%)]			
SIRS	51/105 (48.6)	40/98 (40.8)	0.27
Leukocytosis	50/118 (42.4)	48/114 (42.1)	0.97
Fever	34/106 (32.1)	25/99 (25.3)	0.28
Physician concern of instability	36/105 (34.3)	25/96 (26.0)	0.20

*NOTE*: *P* values were calculated using the Chi square or Fisher’s exact test. *P* value threshold for significance was <0.05 (statistically significant values are bolded). SSTI, skin and soft tissue infection (including cellulitis, necrotizing soft tissue infection, cutaneous and muscular hematomas, ulcers, osteomyelitis and surgical site infections); *SIRS* systemic inflammatory response syndrome

#### Clinical status when repeat cultures were drawn

A total of 28.7 % of patients were febrile at the time of repeat culture (Table 3). Of the patients for whom documentation regarding the physician’s impression of their clinical status was available, only 30.3 % of repeat cultures were drawn to work-up an unstable patient, while the other 69.7 % were surveillance cultures (Table 3). None of the patient demographic or comorbidity characteristics were predictive of persistent bacteremia (Table 3).

#### Antibiotics and source control

The majority of patients had appropriate empiric antibiotic coverage, including those with cleared and persistent bacteremia (Table 4). Only 3 patients, all in the persistent bacteremia group, had the choice of antibiotics influenced by the results of the repeat blood culture. These patients all had line infections with commensal skin organisms, and antibiotics were withheld until repeat cultures demonstrated that the organism was persistent and not a contaminant.

**Table 4:** Nested case control analysis: antibiotic and source control issues for patients with repeat cultures

	Cleared bacteremia (*n* = 118)	Persistent bacteremia (*n* = 118)	*P*
Appropriate antibiotics n (%)			
Empiric	67 (57.8)	72 (61.0)	0.51
Guided by index culture	51 (43.2)	40 (33.9)	0.14
Guided by repeat culture	0	3 (2.5)	0.123
Source control required n (%)	61 (51.7)	74 (62.7)	0.087
*Endovascular*	32 (27.1)	41 (34.7)	0.21
Line removal	28 (23.7)	35 (29.7)	-
PPM/ICD explantation	1 (0.9)	1 (0.9)	-
Valve surgery	3 (2.5)	6 (5.1)	-
Aortic graft replacement	0	1 (0.9)	-
*Spine and Joint*	3 (2.5)	10 (8.5)	0.051
Epidural abscess/spinal	2 (1.7)	5 (4.2)	-
Peripheral joint	1 (0.9)	5 (4.2)	-
*Thoraco-abdominal*	18 (15.3)	13 (11.0)	0.34
Pericardiocentesis	0	1 (0.9)	-
Empyema	2 (1.7)	2 (1.7)	-
Intra-abdominal abscess	3 (2.5)	1 (0.9)	-
Liver abscess	2 (1.7)	1 (0.9)	-
Cholangitis	4 (3.4)	2 (1.7)	-
Peritonitis	1 (0.9)	2 (1.7)	-
Nephrostomy tube	1 (0.9)	2 (1.7)	-
Foley removal	4 (3.4)	2 (1.7)	-
ICP monitor removal	1 (0.9)	0	-
*SSTI*	8 (6.8)	10 (8.5)	0.62
Debridement/abscess drainage	8 (6.8)	8 (6.8)	-
Amputation	0	2 (1.7)	-
Source control done in 48 h n (%)	32/61 (52.5)	22/74 (29.7)	<**0.001**
Endovascular	16/32 (50.0)	16/41 (39.0)	0.35
Spine and joint	2/3 (66.7)	0/10	**0.038**
Thoraco-abdominal	11/18 (61.1)	6/13 (46.2)	**0.046**
SSTI	3/8 (37.5)	0/10	0.069

*NOTE*: *P* values were calculated using the Chi square or Fisher’s exact test. *P* value threshold for significance was <0.05 (statistically significant values are bolded). *PPM* permanent pacemaker, *ICD* implanted cardiac defibrillator, *ICP* intracranial pressure, *SSTI* skin and soft tissue infection

There was no significant difference in the total number of patients with a clinical syndrome that required source control for adequate management (Table 4). For those that required source control, individuals with persistent bacteremia were less likely to have undergone definitive management within 48 h of the index bacteremia (22/74 (29.7 %) vs 32/61 (52.5 %), *p* < .001) (Table 4). This difference was mainly driven by significant delays for spinal/peripheral joint and thoraco-abdominal sources of infection (Table 4).

### Multivariate analysis

Multivariable logistic regression analysis was performed to examine independent predictors of persistent bacteremia (Table 5). Factors that were independently associated with persistent bacteremia included male sex (adjusted OR [aOR] 2.59; 95 % confidence interval [CI] 1.28–5.25), admission to a medical service (aOR 2.80; 95 % CI 1.34–5.84), *S. aureus* bacteremia (aOR 4.49; 95 % CI 1.88–10.73), and endovascular (aOR 7.66; 95 % CI 2.30–25.48) or epidural (aOR 26.99; 95 % CI 1.91–391.08) focus of infection (Table 5).

**Table 5:** Nested case control analysis: multivariate analysis to identify predictors of persistent bacteremia in patients with repeat cultures

Variable	Odds ratio	95 % CI	*P* value
Sex			
Male	2.59	1.28–5.25	**0.008**
Female^a^	1.00	1.00	1.00
Admitting service			
Medical in-patient	2.80	1.34–5.84	**0.006**
Heme-onc in-patient	1.21	0.44–3.33	0.71
Surgical in-patient^a^	1.00	1.00	1.00
Organism			
*S. aureus*	4.49	1.88–10.73	<**0.001**
Other Gram-positive bacteria	0.80	0.35–1.82	0.59
Anaerobes	0.96	0.15–6.38	0.97
All Gram-negative bacteria^a^	1.00	1.00	1.00
Source of bacteremia			
Endovascular	7.66	2.30–25.48	<**0.001**
Epidural abscess/discitis	26.99	1.91–391.08	**0.015**
Other extravascular source	3.02	0.97–9.41	0.057
Unknown source^a^	1.00	1.00	1.00

*NOTE*: Multivariable logistic regression was performed including all variables associated with persistent bacteremia in bivariate analyses in the nested case control analysis, with stepwise backward selection for variable reduction until all retained variables were statistically significant at *p*-value threshold <0.05 (statistically significant values are bolded). Referent categories are indicated with ‘^a^’. *Heme-onc* hematology-oncology in-patient ward

### Costs of repeat culture testing

There were a total of 1620 blood cultures drawn for the patients included in the nested case control analysis during their bacteremic episode. Extrapolating this to all patients with repeat cultures, there were 4130 blood cultures drawn (1131 positive and 1996 negative) at an estimated cost of $105,717–$212,900 [15, 16]. Since only 69.7 % of repeat cultures were done on stable patients, after removing individuals with appropriate indications for repeat cultures (*S. aureus* bacteremia, endovascular or epidural focus of infection, clinical deterioration), there were 1031 inappropriate repeat cultures (134 positive, 897 negative) that could have been avoided, at a cost savings of $22,433–$58,250.

## Discussion

In this retrospective cohort study, we assessed the clinical and microbiological variables associated with the decision to repeat blood cultures for a patient with bacteremia, and with a repeat culture remaining positive (persistent bacteremia). Key associations with persistent bacteremia included endovascular sources of infection and *S. aureus* bacteremia, as well as the inability to achieve source control by 48 h. Cleared bacteremia was associated with *E. coli*, β-hemolytic and viridans group streptococci, and a genito-urinary source of infection.

Our results support those of Lopez Dupla et al. [10], demonstrating a shift in the epidemiology of persistent bacteremia (termed breakthrough bacteremia in their study) since the 1980s from Gram-negative intra-abdominal infections to endovascular infections caused by Gram-positive bacteria. The overall incidence of persistent bacteremia was similar in both studies (6.6 % vs 6.1 %), and associations between persistent bacteremia and microbiologic/clinical variables were comparable [10]. As they speculated, this change in epidemiology likely reflects the increased prevalence of central venous catheters in medical patients, both for acute medical care and chronic management of malignancy and end-stage renal disease [10]. In contrast to their study, we distinguished between bacteremic patients that did and did not undergo repeat cultures. This allowed us to determine the prognostic significance of having a repeat culture drawn, and to assess patterns of repeat blood culture use. We showed that having a repeat blood culture drawn, regardless of the result, was a marker of increased 30-day mortality. This suggests that there are unique characteristics of patients at the time of repeat culture, as determined by the ordering physician, which prompt repeat cultures and portend worse outcome. A similar association has been documented between mortality and having an initial blood culture drawn [17].

The majority of patient and pathogen characteristics associated with persistent bacteremia were also associated with having a repeat culture drawn, which suggests that physicians mostly repeat cultures in situations in which persistent bacteremia is likely to occur. In contrast, variables that were associated with ordering repeat cultures but *not* with persistent bacteremia suggest potential scenarios of overuse of repeat cultures. This pattern was found for surgical in-patients as well as bacteremia caused by β-hemolytic and viridans group streptococci. For surgical in-patients, the tissue trauma and peri-operative stress can itself evoke leukocytosis, fever or SIRS, which may raise concern of infection and prompt unnecessary blood culture use. Previous data show blood cultures to be low yield in certain post-operative settings [18, 19]. The overuse of repeat cultures for streptococci may be explained by the decision to work such patients up for endocarditis with multiple blood culture sets. Our study suggests that such cultures are low yield after the 48-h mark and should be avoided. A better strategy in such cases would be to obtain 3 sets of blood cultures within the first 24 h of admission, preferably prior to antibiotic administration since antibiotics decrease blood culture yield [20, 21].

This study confirms that repeat blood cultures are low yield in most cases of documented bacteremia, particularly in patients receiving antibiotics. While there are few studies addressing persistent bacteremia and repeat cultures, guidelines only recommend repeat cultures in the setting of endocarditis, *S. aureus* bacteremia or if the patient has a clinical deterioration [8, 9]. Fever alone does not necessitate repeat cultures, since patients may remain febrile for days after antibiotics have been started. Persistence of fever was not associated with increased mortality for in-patients with microbiologically identified infections [22]. Clinical judgment is required to decide which patients warrant repeat blood cultures, but our study did not detect any clinical variables (fever, SIRS criteria, leukocytosis) to be predictive of persistent bacteremia at the time of repeat culture. The poor operating characteristics of these variables highlights the need for better markers in the detection and prognostication of severe bacterial infections.

Surveillance cultures are valuable for endocarditis and *S. aureus* bacteremia, since the presence of persistent bacteremia may guide decisions regarding the need for definitive source control. In cases where there is concern for clinical deterioration during treatment for bacteremia, the other role for repeat cultures would be to identify a new bacteremic episode with a pathogen that was not present in the initial blood culture. Grace et al. showed this to be a rare event, occurring in 1 out of 139 (0.72 %) bacteremias [20]. There was a similarly low rate in our cohort, with 26 events (1.4 %) identified. The cause of the increased mortality associated with persistent bacteremia is not clear, but our study showed an association between persistent bacteremia and a failure to achieve source control at 48 h, which is one plausible explanation. Achieving prompt source control may help to clear the bacteremia and decrease mortality, but prospective studies are necessary to address this issue.

Limitations of our study include its retrospective design, which prevented us from directly ascertaining the indication for repeat culture testing, as well as the small number of cases of persistent bacteremia, which limited our ability to detect more subtle predictors of persistence. Blood culture collection practices and blood culture yield may vary in hospitals with different thresholds for testing and labs using different culturing media or techniques. The fact that this is a single-centre study could limit the application of our results to other settings, however the fact that the detected associations with persistent bacteremia are consistent with previous literature suggests that our findings are not unique to our institution. The reliance on physician documentation for aspects of the patient’s clinical status and the infectious syndrome likely lead to an over-classification of patients into the “unknown source” category. Due to the multiple comparisons in our analysis, there is a risk of false-positive inferences. However, due to the exploratory nature of the study, we opted not to apply an adjustment for multiple comparisons [23]. Cost calculations were subject to a number of assumptions as well as the use of pricing data not specific to our institution, and thus we opted to report a range that is likely to encompass the actual amount. Finally, while this study assesses the incidence and prognostic value of persistent bacteremia, we cannot infer whether its detection improves outcomes (i.e. mortality/length of stay), or alters therapy (i.e. antibiotics choice/duration, timing of source control).

## Conclusions

Repeat blood cultures have utility in assessing patients at risk of persistent bacteremia, particularly those with *S. aureus* bacteremia and endovascular source of infection. Ensuring that source control is achieved within 48 h of the index bacteremia may reduce the risk of persistence. Whether this also reduces the excess mortality associated with persistent bacteremia should be studied further. Common situations in which repeat blood cultures offer low yield include bacteremic genito-urinary infections, and bacteremias caused by Gram-negative bacteria, viridans group and β-hemolytic streptococci.

## Abbreviations

aOR, adjusted odds ratio; CI, confidence interval; Heme-One, hematology/oncology in-patient ward; HIV, human immunodeficiency virus; HL7, health level 7; ICD, implanted cardiac defibrillator; ICP, intracranial pressure; ICU, intensive care unit; IQR, interquartile range; PPM, permanent pacemaker; SHSC, Sunnybrook Health Sciences Centre; SIRS, systemic inflammatory response syndrome; SPIRIT, Stewardship Program Integrated Resource Information Technology; SSTI, skin and soft tissue infection (including cellulitis, necrotizing soft tissue infection, cutaneous and muscular hematomas, ulcers, osteomyelitis and surgical site infections).
